# The Frailty Syndrome: An Emergent Property of Parallel Dysregulation in Multiple Physiological Systems?

**DOI:** 10.1093/geroni/igab046.1482

**Published:** 2021-12-17

**Authors:** Alan Cohen, Ahmed Ghachem, Linda Fried, Véronique Legault, Karen Bandeen-Roche, Nancy Presse, Pierrette Gaudreau

**Affiliations:** 1 Universite de Sherbrooke, Sherbrooke, Quebec, Canada; 2 Mailman School of Public Health, New York, New York, United States; 3 Université de Sherbrooke, Sherbrooke, Quebec, Canada; 4 Johns Hopkins Bloomberg School of Public Health, Baltimore, Maryland, United States; 5 Université de Montréal, Montreal, Quebec, Canada

## Abstract

Despite its widespread presence in older adults, frailty etiology is still unclear, being associated with dysregulation in diverse physiological systems. Here, we show evidence that frailty emerges from broad loss of homeostasis integrated through complex systems dynamics. Using the NuAge and WHAS cohorts, we calculated Mahalanobis distance-based physiological dysregulation in six systems and tested the breadth, diffuseness, and nonlinearity of associations between frailty and system-specific dysregulation. We found clear support for breadth of associations, but only partial support for diffuseness and nonlinearity: 1) physiological dysregulation is positively associated with frailty in many or all systems, depending on analyses; 2) the number of dysregulated systems or the total amount of dysregulation are more predictive than individual systems, but results only partially replicated across cohorts; 3) dysregulation trends are exponential, but not always significant. These results suggest, but do not fully prove, that frailty is an emergent property of complex systems dynamics.

